# Odontological Society of Pennsylvania

**Published:** 1898-08

**Authors:** 

**Affiliations:** Odontological Society of Pennsylvania


### Reports of Society Meetings.

### ODONTOLOGICAL SOCIETY OF PENNSYLVANIA.

A STATED meeting of the Odontological Society of Pennsylvania was held at 1415 Walnut Street, Philadelphia, February 12, 1898, at eight P.M., President Brubaker in the chair.

In the regular order of business the chair appointed Drs. Broomell and Warren as a committee to take action on the death of our esteemed member, Dr. Alonzo Boice.

A paper on "Empyema of the Antrum" was then read by Dr. Ella E. Musson.

(For Dr. Musson's paper, see page 496.)

### DISCUSSION.

*Dr. Cryer.*—I am interested in the subject of Dr. Musson's paper, as it is closely related to the studies I have been making for the last few years upon the relation of the teeth with the floor of the maxillary sinus, the shape of the sinus and its outlet into the nasal chamber, and the relation of the outlet with the frontal sinus through the hiatus semilunaris.

The essayist has shown specimens and illustrations which do not agree with the general text-books upon anatomy. Anatomy is generally taught as an exact science in our books and by teachers, but from the special or surgical point of view it cannot be properly so classed. I do not mean by this that there is not such a regular basis of anatomical science that certain rules and types cannot be made and followed, but the more closely we study the subject the more variations as to detail we are compelled to record. There are as many variations in the internal anatomy of individuals as there are differences in their external appearances; especially is this true concerning the anatomy of the head, modified as it is by age, disease, occupation, climate, race, and other conditions. I can certainly agree with what the essayist has said in speaking from her own experience, either from clinical observation or her own anatomical dissection, but I do not agree with many of her quotations, among which may be mentioned the following:

Hajck thinks the pain in acute abscess of the antrum "is greatest with a thin floor, as the dental nerves are thus in close relation to the mucous membrane of the sinus." In normal anatomy the superior dental nerves and vessels pass in a groove which extends from the internal opening of the posterior dental foramen horizontally to the anterior portion of the sinus. The groove terminates into a canal or canals through which the nerves and vessels pass to the anterior teeth and surrounding tissue. From this groove branches are sent out for the accommodation of nerves and vessels going to the roots of the various teeth in this region: occasionally these grooves may be lost in small canals for a short distance. The nerves and vessels are only covered by the muco-periosteum. Any disease of the antrum, whether the floor be thick or thin, is liable to interfere with the normal function of these nerves and vessels.

I must also take exception to the statement that "Gavel diagnoses antral abscess by syringing it out through the ostium maxillare, having succeeded in passing the catheter in twenty-eight cases out of forty." The statement that an irrigating catheter is so passed may sound or read well, but in actual practice it is out of the question. It cannot be passed in normal cases. Of course there are cases where a catheter can be passed from the nose into the maxillary sinus, for at times there are abnormal openings, either pathological or traumatic, and even the greater portion of the division between the antrum and the nose has been lost in some cases. In these there is no difficulty in washing out the sinus.

The method of Dr. Moll, given at the last annual meeting of the Dutch Laryngological Society, consists of the "aspiration of the contents of the accessory cavities by a forcible inspiration, while the mouth and nose are firmly closed. In this way the chest is inflated, and there is considerable diminution of pressure in the accessory cavities." Dr. Moll may, by some mysterious method, be able to inflate his chest with his mouth and nose closed. It would be interesting to have the exact technique of this method made public.

The essayist speaks of a large antrum on one side associated with a small one on the other. To account for this and other variations it is necessary to understand the development and the growth of the sinus.

The maxillary sinus varies in shape, size, and thickness of its walls according to age, race, and presence or absence of teeth and tooth-germs within the jaw of each individual. The capacity varies in different subjects, and in the two bones of the same subject. The development of the sinus begins about the fourth month of gestation by an invagination of the lining membrane of the nose within the hiatus semilunaris. At the time the invagination begins, and until the eruption of the permanent teeth, the greater portion of the axilla is occupied by the dental organs. As the invagination progresses the cancellated portion of the bone undergoes absorption. This absorption of the internal portion of the maxilla is continued in a variable degree throughout life, until, in old age, the walls usually become exceedingly thin. Usually the floor of the antrum in the negro race is much thicker than in the white race, and it is uncommon to find the points of the roots covered only by a thin plate of bone as in the white race. If the intrinsic forces normally related to and operating upon the developing jaws be not interfered with, there will be a normal development of the bone, the sinuses and structures associated with it. But if this harmonious relation of form and structure be disturbed, there will be a corresponding abnormal form to the bone, sinuses and structures associated with it, and in degree proportional to the force applied. This abnormal force can be manifested in various ways,—the lack of proper nourishment and assimilation to the various tissues involved. Diseases of the soft parts often change the shape of bones, particularly in the young. Aggravated tonsillitis on one or both sides in childhood will, if chronic and accompanied by hypertrophy, cause the roof of the mouth to take an inverted V-shape, and this will influence the shape of the floor and the size of the maxillary sinus. Inflammation in and about the deciduous teeth will induce a vascular excitement that in some cases causes the osteoblasts to deposit secondary bony tissue through the cancellated tissue, thus interfering with the proper eruption of the permanent teeth either by causing their impaction or their eruption in an abnormal position. A slight disarrangement in early life will cause variations that cannot be overcome at a later period.

I have specimens here that are almost like the drawing shown by the essayist. On one side the antrum extends far below the level of the floor of the nasal cavity, while on the other it does not extend to the level of the floor of the nasal chamber. The nasal chamber on this side extends outward to the external wall of the maxillary bone.

The outlet of the antrum, though not generally so described, is on the superior edge of the inner wall of the sinus, a little anterior to the centre of the edge. It is oval in shape, and permits communication between the sinus and the nasal fossa through the hiatus semilunaris. In many skulls this opening is in direct communication with the frontal sinus and the middle and anterior ethmoidal cells. Occasionally the opening of the sinus is in the roof of the antrum. There are skulls where a probe can be passed directly from the sinus through the hiatus semilunaris into the frontal sinus.

I was in hopes that Dr. Musson would speak of the bulla ethmoidalis. It is situated on the upper portion of the lateral wall of the hiatus semilunaris, and extends upward and inward towards the uncinate process. It contains the middle ethmoidal cells, varying in number and size. Their openings are in the outer and lower portion of the bulla ethmoidalis, and discharge their fluids into the hiatus semilunaris near the opening into the maxillary sinus. It is through the increase in size of the bulla ethmoidalis and of its covering membrane that much disturbance is caused in the anterior and posterior portion of the nasal chambers, the frontal sinuses, and antrum of Highmore.

By its enlargement it presses towards or against the septum, closing the space. When the enlargement is downward it presses upon the unciform process and into the hiatus semilunaris, closing this semicircular groove and preventing the passage of fluids from the frontal sinus and the anterior and middle ethmoidal cells into the posterior portion of the middle meatus, consequently the fluids enter the maxillary sinus, and, through general inflammation of these parts, there is an excess of these normal fluids that cannot find exit. This would interfere with the proper nourishment of the teeth, especially in the white race, and, as has been already stated, the nerves and blood-vessels to the teeth pass on the inside of the outer wall of the sinus. The excess of fluid without an exit will cause a fulness of all the anterior cells as well as the frontal sinus, and even cause disturbances in the anterior portion of the brain-case. It is this thin blocking of the hiatus semilunaris which causes the secondary or associate opening between the antrum and nasal chamber, which is always found posterior or below the bulla ethmoidalis. I have many cases on record where teeth have been lost by discharges of infected fluids from the antrum along the roots into the mouth.

*The President.*—The difficulties and complexities attending the treatment of antrum troubles certainly become very apparent. There is a similar but not identical condition of the antrum which might be termed emphysema, which has a great interest for dentists, inasmuch as the cause of it is to be found in the conditions of the teeth which project into the antrum. I will ask Dr. Kyle to speak on that phase of the subject.

*Dr. Kyle.*—Mr. President, ladies, and gentlemen, I am sorry that I did not hear the entire paper of Dr. Musson on "Empyema of the Antrum." That is one subject at least on which the dentist and the rhinologist are on the same ground. Pus in the antrum differs not from pus in any other part of the body, and if you drain from below the case passes out of the hands of the rhinologists and into the hands of the dentists. I think sometimes we make a mistake in draining from above.

The condition of emphysema of the antrum,—I do not know whether it is a new condition, but I have noticed the symptoms were the same as of empyema. In several cases it was associated with ozæna, in which there was no accounting for ozæna. The ozæna was intermittent. At times it would disappear. In one case I did not refer to the dentist, but opened and found no pus; but there was an escape of gas. There was a fetid odor. In keeping the antrum open the ozæna would disappear. The gas comes from decomposition of diseased tissue. You know that even in decomposition of tissue exposed there is gas formed. And when tissue is decomposing in a closed space there must be an escape for that gas; and that would lead to emphysema of the antrum. In one case where the patient suffered very much, as in empyema of the antrum, the dentist found the tooth was dead, and there was considerable necrosis; and in passing the probe up into the antrum he did not strike pus, but this fetid odor, and after this the symptoms all disappeared. In that case emphysema of the antrum was relieved by the treatment of the tooth. It gives rise very much to the same symptoms as pus. I believe that in five of the six cases on which I have notes I was wrong in my diagnosis of pus in the antrum; it was due to a lesion in the tooth rather than from any necrotic process. I would like to hear if the dentists have noticed anything similar.

*Dr. Peirce.*—In listening to the very interesting paper of Dr. Musson and the discussions that followed, I am inclined to think that all of my knowledge and practice is largely empirical. I can readily remember the first opening that I made in the antrum. While having charge of a clinic in 1857, a patient came in with a large tumor under the left wing of the nose. I probed it with a sharp instrument, and found it was a curved root presenting itself under the lobe. I took hold of the root with a forceps and brought out a root of a large incisor tooth, which penetrated the antrum. This extraction was my first experience with the antrum.

Only a few days following a lady presented herself, who said that for some time she had not gone into a stage-coach or parlor without the inmates putting handkerchiefs to their noses. I found she had had a molar tooth extracted, and had had some pain on that side of the face. I made the opening free into the antrum, and found by washing it out that there was considerable pus. By washing a few times she was relieved, and such was the result that some time afterwards when I met her husband he said, "Doctor, I had a very disagreeable wife, and you have given me a very pleasant one."

Since that time I have opened and treated a great many antra. I have always gone into them either over the alveolar ridge or, if I have selected a tooth, the anterior buccal surface of the molar. I have not disturbed the teeth other than treating them.

I had an interesting case this week of a tooth opening into the nares. A physician came to me a few years ago with a broken incisor. I placed on a crown, and it was comfortable for some time, but finally he said that there was a discharge from that tooth. The discharge has continued for six or eight months, and this week the tooth dropped out, leaving an opening from the apical extremity right through to the nares. The trouble ceased at once. I never have treated nor have any knowledge of diseased antra except in treating the teeth.

My friend Dr. Cryer says that there is always a process of bone over the root, but when a tooth has an opening at the apical extremity the tooth will very soon cause the absorption of that very thin bone. The product of decomposition from this dead pulp is emptied into the antrum directly, and produces no fistula into the mouth. And this gives rise to a diseased condition of the mucous membrane in the nostrils and causes an unpleasant odor. I am inclined to think that a large proportion of diseased antra have come from the teeth. And even though the teeth have been extracted before the diseased antrum goes into the hands of the physician, a diseased tooth has probably been responsible for the condition. That has been my experience, and, I think, the experience of other dentists.

*Dr. Cryer.*—I would like to answer Dr. Peirce in regard to the absorption of the thin plate of bone that covers a portion of the roots of teeth in the floor and on the sides of the antrum. In the white race the roots of the molar teeth are closely associated with the antrum; there are specimens upon the table illustrating this. It is generally admitted that pus or infected matter will pass in the direction of the least resistance, and by observation it would appear that when infected matter, such as pus, passes out of the apical foramen of the teeth, it would more than likely pass into the antrum instead of through the alveolar process into the mouth. My clinical experience teaches me otherwise,—*i.e.*, an abscess caused from an infected tooth usually makes its fistula into the mouth. The only reason that I can give for this is that nature usually protects weak points in various ways. In this case I believe the mucoperiosteum lining of the sinus takes up its defence, and through its osteoblastic layer builds and protects its wall against invasion through absorption. I have here the right and left superior maxillæ of the same skull, each having a devitalized first molar; abscesses have taken place at the apex of their palatal roots. In the left maxilla the opening has been made into the maxillary sinus, but there is evidence that there has been an attempt to build bone to prevent its doing so. In the right maxilla there is quite an exostosis over the position of the infected root, and by examination of the palatal surface of the root it is found that the discharge of the abscess was made into the mouth, and I believe that is the usual outlet for the apical abscesses, of course recognizing that they occasionally open into the antrum. I believe that diseased antrum more frequently comes from malformed and diseased nasal chambers than from the teeth.

*Dr. Warren.*—There are one or two points I think might be emphasized in the treatment of this disease. One is the matter of opening at the lowest point. I would like to ask Dr. Kyle why he does not always open at the lowest point. The second is the matter of drainage. In many cases we find openings made by surgeons or specialists that are packed with gauze; others will use ivory plugs and vulcanite, and occasionally we hear of a drainage-tube. During the last three or four years I have opened a number of diseased antra, both for others and myself, and have found in every case where I have treated them that I secure good results with free drainage obtained by introducing drainage-tubes. These tubes should be so constructed as to be capable of being sterilized and flushed by the patient or some member of his family at least once a day. I use the small sword-shaped drill, following that with a small Morse reamer, and then have my platinum tube drawn to a size that would fit the opening made by the last drill.

*The President.*—If there is any one else who wishes to speak upon this subject we will be very glad, indeed, to hear from them.

*Dr. Cryer.*—There is a tendency, however, on the part of some of our rhinologists to make outlets from the mouth into the maxillary sinus, thus giving passage to the fluids, diseased or otherwise, that have passed from the superior anterior portion of the nose and the frontal sinus into the antrum through their inability to make them pass along their normal course. This is not good surgery. The mouth must be kept pure and clean from these foul discharges, and it is the duty of those who care for the nose to treat and cure their special region without infecting the oral cavity.

*Dr. Musson.*—As to the remarks of Dr. Cryer, I think Zuckerkandl's work does not deserve to be considered antiquated. It dates back only some ten years or more, and the illustrations are all taken from anatomical specimens. They are all plates of conditions as he saw them there. I do not know of any illustrations as beautiful as those of Zuckerkandl. It is possible Dr. Cryer can tell me of some others. Bodenheim's work dates only a few years back. In regard to the position of the ostium maxillare, in all these specimens I have shown to-night they have been up very high on the naso-antral wall, and not low down. The opening that is probed down the middle meatus is generally an accessory opening. As to the probing of the antrum, I have never succeeded but once in doing it. It is often impossible unless, of course, the middle turbinate is removed.

As to the pressure, I think Dr. Cryer hardly did Dr. Moll's method justice. I said both the nose and mouth were tightly closed. Then by expanding the lungs the atmospheric pressure is considerably lowered, and it is possible to draw the liquids up and down.

In 1882, I think, the opening of the frontal and ethmoidal sinuses into the antrum were shown by Mackenzie and Macdonald, of King's College, London. The interesting point in regard to the accessory opening of the antrum is the explanation that these accessory openings are only found in rather old people and due to absorption.

It was Dr. Peirce himself who first taught me that opening through the canine fossa. Of course, I do not know that it is advisable except in a case of long standing. It seems a pity to sacrifice a tooth.

Now, as to the drainage-tubes of Dr. Warren. There are two objections. First, they slip around in the canal, and they tend to cause absorptive abscess. These objections have been overcome by French dentists. They fasten it with bands to the teeth, anteriorly and posteriorly, and a certain amount of fixity is produced. Another objection is that unless one is quite sure of the length he is likely to get it a little too long. If the tube is kept in position while washing a certain amount of pus remains about the end of it. If the tube is too long it is hard to thoroughly wash out the pus. In making the drainage-tubes I have always been careful to pass a probe and mark it off, and have the drainage-tube made of that length.

*Dr. Peirce.*—I would like to ask Dr. Musson whether persons seem to inherit from their ancestors a tendency to disease of the antrum. Four sisters have been in my hands, all of whom have suffered from this trouble.

*Dr. Musson.*—I have not noticed it.

*Dr. Warren.*—Just a word in reply to the doctor's criticism. Of course we always take the measure. In making the perforations we have the lower row enlarged. We are always careful to explore the cavity and mark the probe, and in that way lay out the work. After marking the probe we make it a little longer. As to the movement of the tube, if the suggestion is followed as I made it,—to use the Morse twist drill,—we find that with some little pressure the tube will go to its place, when, if necessary, we can tie it to the teeth. But I have never experienced that difficulty.

*Dr. Bonwill.*—I did not hear what Dr. Musson had to say, but I read her paper. I think it is very admirably done. I would like to ask the doctor whether she, so young as she is, has had all these cases herself,—whether or not they come from her own practice or from the practice of the dentists surrounding her? for in a practice of forty-three years I have never had but one single case in my own patients, and that never happened until after the tooth had been treated,—thirty-five years ago. I placed a crown on it twenty years ago, and that crown I had to remove about two years since. Three months ago I extracted the tooth, and I found an opening into the antrum.

I have had a few cases from the hands of others. I think many are due to the stupidity of dentists, and a great deal of it might be obviated. The great thing, when you see a trouble, is to extract a tooth without waiting. This is very largely the practice that I would recommend.

*The President.*—It has been moved and seconded that we adjourn.

Adjourned.

JOSEPH HEAD, M.D., D.D.S.,
*Editor Odontological Society of Pennsylvania.*

